# Supplementary material for: A Global Screen for Assembly State Changes of the Mitotic Proteome by SEC-SWATH-MS
Source: Cell Syst. 2020 Feb 26;10(2):133–155.e6. doi: 10.1016/j.cels.2020.01.001 (PMC7042714; doi:10.1016/j.cels.2020.01.001)

P46939 | UTRO_HUMAN | UTRN DMDL DRP1
Monomer MW [kDa]: 394.466 Monomer expected elution fraction: 28

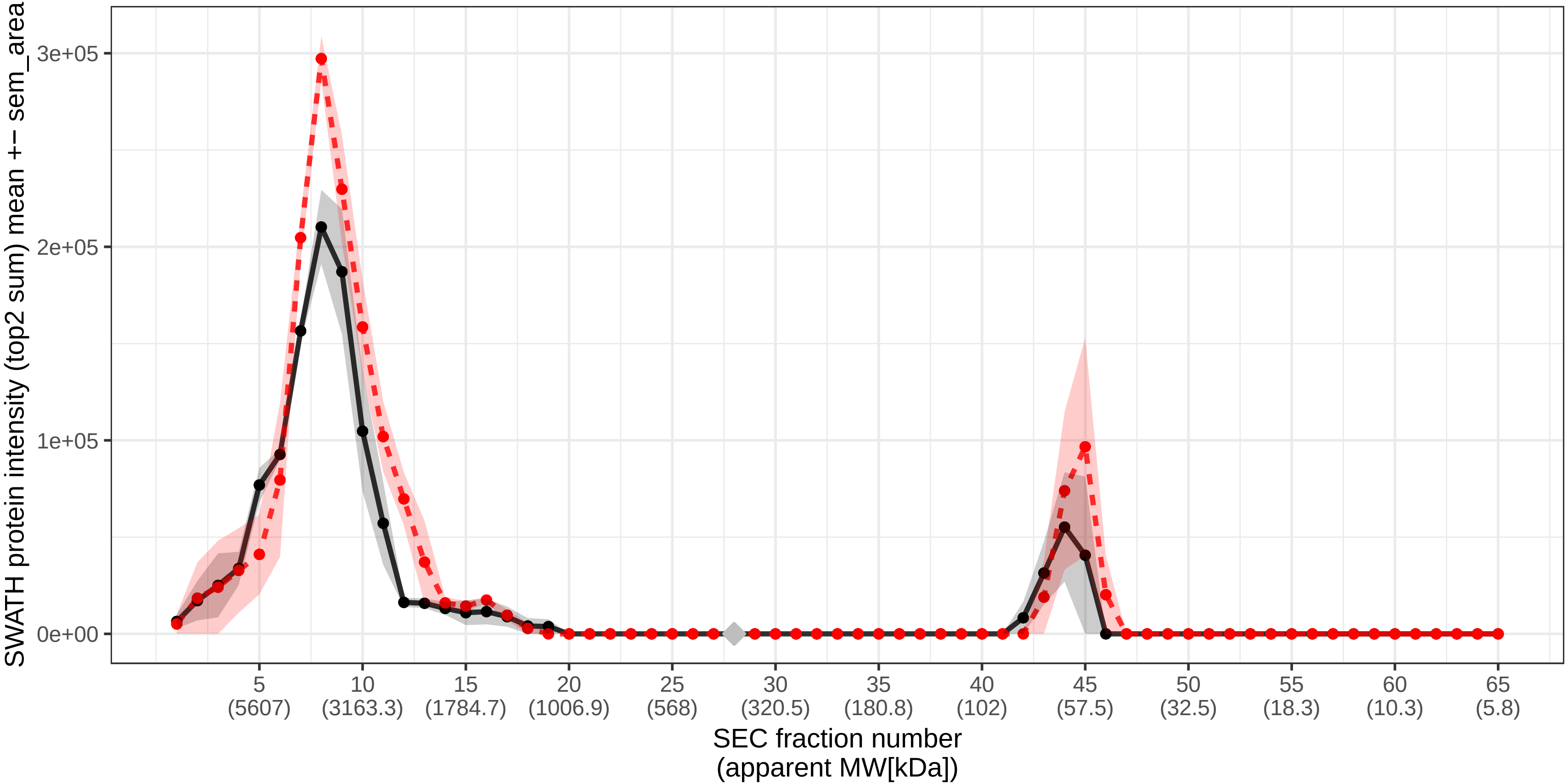

Supplement: Data S1. SEC-SWATH-MS Protein Chromatograms, Related to Figure 1 [file mmc6.zip › SECchrom_P46939_UTRO_HUMAN_UTRN_DMDL_DRP1.pdf]
